# The use of Fourier‐transform infrared spectroscopy to characterize connective tissue components in skeletal muscle of Atlantic cod (*Gadus morhua* L.)

**DOI:** 10.1002/jbio.201800436

**Published:** 2019-07-01

**Authors:** Karen W. Sanden, Achim Kohler, Nils K. Afseth, Ulrike Böcker, Sissel B. Rønning, Kristian H. Liland, Mona E. Pedersen

**Affiliations:** ^1^ Nofima AS Ås Norway; ^2^ Faculty of Science and Technology Norwegian University of Life Sciences Ås Norway

**Keywords:** chondroitin sulfate, collagen, connective tissue, FTIR micro spectroscopy, proteoglycans

## Abstract

In the present study, Fourier‐transform infrared spectroscopy (FTIR) is investigated as a method to measure connective tissue components that are important for the quality of Atlantic cod filets *(Gadus morhua L.)*. The Atlantic cod used in this study originated from a feeding trial, which found that fish fed a high starch diet contained relative more collagen type I, while fish fed a low starch (LS) diet contained relative more glycosaminoglycans (GAGs) in the connective tissue. FTIR spectra of pure commercial collagen type I and GAGs were acquired to identify spectral markers and compare them with FTIR spectra and images from connective tissue. Using principal component analysis, high and LS diets were separated due to collagen type I in the spectral region 1800 to 800 cm^−1^. The spatial distribution of collagen type I and GAGs were further investigated by FTIR imaging in combination with immunohistochemistry. Pixel‐wise correlation images were calculated between preprocessed connective tissue images and preprocessed pure components spectra of collagen type I and GAGs, respectively. For collagen, the FTIR images reveal a collagen distribution that closely resembles the collagen distribution as imaged by immunohistochemistry. For GAGs, the concentration is very low. Still, the FTIR images detect the most GAGs rich regions.

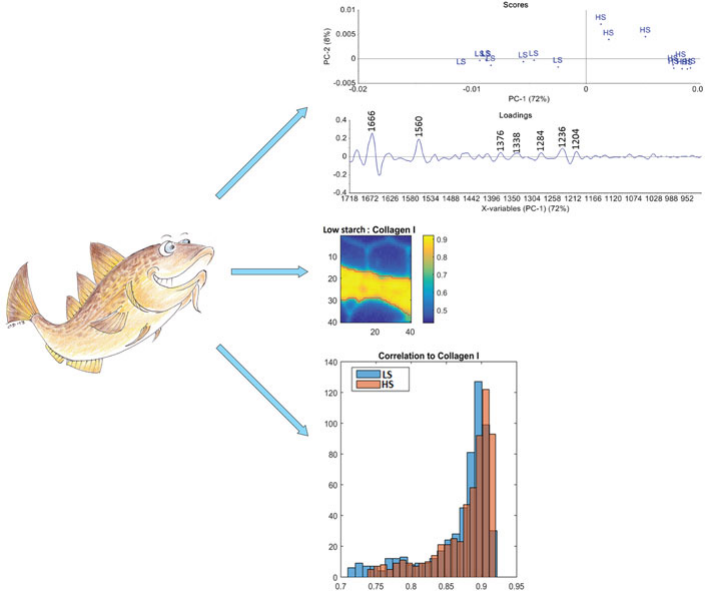

## Graphical abstract

Fourier‐transform infrared spectroscopy (FTIR) micro and imaging spectroscopy is used to analyze the relative amount of two important components, collagen and proteoglycans in connective tissue in cod. The cod received two diets with different amount of starch. The spectra obtained from the connective tissue is compared with pure commercial components, collagen I and chondroitin‐4‐sulfate. Principal Component Analysis and FTIR correlation images could separate the two diets well according to collagen I.

## 1 INTRODUCTION

Fish filet quality is an important factor for consumer acceptance. Gaps, tears and splits in filets are major problems for the fish industry today. It is well known that composition of the connective tissue surrounding the muscle fibers is one of the main causes of these quality defects, as gaps occur when connective tissue fails to hold the filets together at the myofiber‐myocommata attachments and between myofibers 1. Connective tissue consists of a complex three‐dimensional structural network of collagens, proteoglycans (PGs) and glycoproteins and has a major role in the attachment of muscle fibers and regulating tissue strength 2. Several types of collagen are the main constituents of connective tissue and they all contain a characteristic three polypeptide chain, which forms into a unique triple helical structure 3. Collagen determines the connective tissue strength and structure 4, and the most abundant collagen type in fish muscle is the fibrillar collagen type I 5, 6. PGs are complex and multifunctional molecules consisting of a protein core with a variable number of covalently attached carbohydrate side chains, the glycosaminoglycans (GAGs) 7. They are important for the spatial organization of collagen fibers, regulating collagen fiber diameter 8, 9 and collagen crosslinking 10, and thereby indirectly the strength of the connective tissue. The pyrodinoline crosslinks in the connective tissue play a key role for the textural properties 10, 11. Their unique carbohydrate parts, the GAGs, consist of a highly linear unbranched polysaccharide of repeating disaccharide units of an amino sugar (N‐acetylglucosamine or N‐acetylgalactosamine) and uronic acid (glucuronic acid or iduronic acid) or galactose which is modified by sulfation. A common GAG in the connective tissue is chondroitin sulfate usually carrying one sulfate group per disaccharide, predominantly in the fourth (C‐4‐S) or sixth (C‐6‐S) position of the hexosamine residue. The spatial distribution of C‐4‐S and C‐6‐S within the connective tissue gives differences in negative charge density. An increased charge density of the GAGs is related to stronger protein binding 12, 13. Previous studies on GAGs in fish muscle have revealed differences in GAG sulfation pattern between species with different gaping properties 14. Non‐gaping species like spotted wolfish (*Anarhichas minor*), have shown to contain 3 to 4 times more sulfated components in the filets than the gaping species such as Atlantic cod 15. The sulfate carrying PG aggrecan has shown a higher amount in hard muscle compared with soft muscle in salmon 16. The hard textured muscles are less prone to water release 17, and aggrecan may play a role here because of good water‐binding properties. Aggrecan is also shown to play a protective role in preventing degradation of collagen fibrils 18.

The composition of connective tissue dictates its mechanical properties. Traditional biochemical methods are used to characterize the components in tissue, and standard histological methods are used to quantify their spatial distribution. These techniques are time‐consuming and often not suitable for obtaining information without extracting components from the material. Chemical analysis results in complete destruction of the tissue, and the native structure disappears.

Fourier‐transform infrared spectroscopy (FTIR) micro/image spectroscopy is a promising tool to gather information on the composition of biological materials 19. Spectroscopic techniques are often fast, more environmentally friendly and provide a large amount of information. The coupling of an FTIR spectrometer to an optical microscope allows to identify and quantify the relative amounts and distribution of chemical compounds in a tissue. The wavelengths of many IR absorption bands are characteristic of specific types of chemical bonds, and the molecular structure information of the components is identified due to their position 20. FTIR imaging can therefore be used to visualize the spatial distribution of biochemical components. In biological and medical science, FTIR microspectroscopy and FTIR imaging spectroscopy have been extensively used to differentiate between diseased and healthy tissues 21. Furthermore, FTIR has been used to monitor for GAGs and collagen content in articular cartilage 22, 23, 24, 25, 26, 27, 28, 29, 30. In these studies, baseline‐corrected spectra are used to quantify collagen content and GAG by the amide I peak intensity and peak intensities in the carbohydrate region, respectively 23, 24. The ratio of the integrated area of the carbohydrate region to the amide I was also used to obtain relative quantities and distribution of the collagen and GAG components 30. However, infrared absorbance bands from different molecules in biological tissue have significant overlap. In particular, the amide I band, which arises mainly from C=O stretching vibration 31, is present in both proteins and GAGs. To enhance the spectral resolution and to reveal the underlying features that are not recognizable in the original spectra, the second derivative can be applied. The second derivative permits a more specific identification of small and neighboring peaks 32, 33 and removes the baselines 34. By the use of second derivative more specific collagen and PG peaks have been located 33, 35 For estimating the spatial PG content, the second derivative peak at 1062 cm^−1^ has shown the most promising results 27.

FTIR spectroscopy is well established in the medical field, but its applicability on connective tissue in fish muscles is less explored. The current study investigates the potential of FTIR spectroscopy to measure connective tissue components, collagen and GAGs in Atlantic cod filets. The Atlantic cod used in this study originated from a feeding trial 36, and by mRNA it was found that fish fed a high starch (HS) diet contained relative more collagen type I. By applying high‐performance liquid chromatography the fish fed the low starch (LS) diet was found to contain about 40% more GAGs in the connective tissue than the fish fed the HS diet.

In this study, FTIR spectra of pure commercial collagen type I and GAGs were acquired to identify spectral markers and compare them with FTIR spectra from connective tissue. As connective tissue distribution differs within a sample, the spatial distribution of collagen type I and GAGs was further investigated by FTIR imaging in combination with immunohistochemistry.

## 2 MATERIALS AND METHODS

### 2.1 Pure commercial components

FTIR spectra of pure commercial collagen type I from human placenta Bornstein and Traub Type I (C7774; Sigma‐Aldrich CO, Germany) and chondroitin‐4‐sulfate (C‐4‐S) from bovine trachea (Sigma‐Aldrich CO) were acquired with FTIR. The purified compound (1 mg) was mixed with a 0.1 M Tris‐ HCl buffer pH 7.4 (1 mL) and homogenized manually. Three replicates of 5 μL of each homogenized mixture were put on a 96‐well Si‐micro plate. The plates were left to dry and stabilize at room temperature over night before measurement.

A mixture of 50% collagen type I and 50% C‐4‐S and a mixture of 90% collagen type I and 10% C‐4‐S was manually homogenized and measured by FTIR.

### 2.2 Atlantic cod muscle preparation before analysis

The Atlantic cod used in this study originated from a feeding trial done by Tingbø 36. The Atlantic cod was (*Gadus morhus L*.) raised at the Aquaculture Research Station outside Tromsø Norway. The fish was handled in accordance with the national rules and regulations and all efforts were undertaken to minimize stress and suffering of the fish. The fish was kept in parallel tanks (70 fish per tank) and were fed the experimental diets for 13 weeks. The fish received two different diets, one with 6.6% starch (LS) and the other with 11.9% starch (HS). To achieve a similar carbohydrate content in both diets, complex fibers were added to the LS diet. Both diets contained approximately 15% carbohydrates. The amount of protein and fat was similar in the diets 36. Sampling was carried out after starving the fish for 48 hours, blood glucose was measured using iSTAT apparatus (Abbot Laboratories, IL), and muscle tissue samples were collected immediately after slaughter. The muscle tissue samples were taken from the area at the base of the dorsal fin. They were cut out with an approximate size of 1 cm × 1 cm × 0.5 cm. The muscle samples were embedded in O.C.T compound (Tissue‐Tek Electron Microscopy Science, Hatfiles), snap‐frozen in liquid nitrogen and stored at −80 °C until use. For FTIR spectroscopy and immunohistochemistry, parallel sections were sectioned transversely to the fiber direction on a cryostat (Leica CM 3050; Leica Microsystem Wetzlar, GmbH, Heidelberg, Germany). The sections were 10‐μm thick and were placed on a ZnSe window for FTIR measurement and a glass slide for immunohistochemistry.

For FTIR spectroscopic analysis and immunohistochemistry, three replicates from three different fish from each diet were selected based on their blood glucose content after slaughter. The content of blood glucose was between 3.2 and 4.1 mmoL/L for the LS fed fish and 9 and 11 mmoL/L for the HS fed fish.

### 2.3 FTIR spectroscopy

FTIR measurements of the pure components were performed using a High Throughput Screening eXTension (HTS‐XT) unit coupled to a Tensor 27 spectrometer (both Bruker Optik GmbH, Germany). The measurement of the microplate was done in transmission mode in the spectral region from 4000 to 750 cm^−1^. Before every sample measurement, a background was collected to account for variation in water vapor and CO_2_. A spectral resolution 4 cm^−1^ was used, and for each spectrum 40 scans were acquired. The acquisition of spectral data was performed by using the OPUS LAB v 6.1 software.

The FTIR microscopic spectra and images were acquired with a Perkin Elmer Spotlight 400 FTIR imaging system (Perkin Elmer, Shelton, CO). It consists of an FTIR spectrometer, an optical microscope and a computer‐controlled sample stage. Single element absorbance spectra and images were recorded in the range from 4000 to 750 cm^−1^ using a mercury cadmium telluride (MCT) detector, with a spectral resolution of 4 cm^−1^. For each pixel, 32 scans were obtained. The pixel size in FTIR images was 6.25 μm. The microscope was sealed with a custom‐made box, and both microscope and spectrometer were purged with dry air to reduce the spectral contribution from water vapor and CO_2_. A background spectrum/image of the ZnSe substrate was recorded before each measurement.

### 2.4 Immunohistochemistry

Immunostaining was performed with primary monoclonal antibodies from mouse for C‐4‐S (mab2030, Millipore) and polyclonal rabbit for collagen (ab20033, abcam). The antibody for C‐4‐S has previously been used to stain for GAGs in tissue samples from cod 37. Secondary antibodies used were obtained from mouse (A11001, Invitrogen) and rabbit (211‐505‐1090, Jackson ImmunoResearch). Sections were fixed with ice‐cold acetone and permeabilized using 0.5% Triton X‐100 in PBS for 15 minutes. To generate the antigenic epitopes, the sections were digested with chondroitinase ABC lyase (cABC) from Proteus vulgaris (0.5 units/mL) in 0.1 M Tris–HCl buffer, pH 8. After cABC treatment for 2 hours at 37°C, nonspecific binding was blocked by using 5% non‐fat dry milk powder dissolved in 1× PBS. Primary antibodies (1:100 diluted) in 2% non‐fat dry milk in PBS were added and incubated overnight at 4°C before washing with PBS for 30 minutes. Subsequent incubation with secondary antibodies was performed for 2 hours, washing with PBS for 30 minutes before using Dako fluorescent mounting medium (Glostrup, Denmark). Negative controls were incubated with secondary antibodies only. The slides were examined by fluorescence microscopy analysis (ZEISS Axio Observer Z1 microscope, Jena, Germany).

### 2.5 Data analysis and image preprocessing

Data analysis of FTIR spectra was performed using the Unscrambler X (version 10.2 and 10.4.1, Camo Software, Norway). The raw spectra were subjected to Savitzky‐Golay second derivatives (9 pt window width, second order polynomial) and basic extended multivariate signal correction (EMSC) (second order polynomials) to remove multiplicative and independent wavenumber baselines. The same pre‐processing was applied to a dataset of reference spectra of C‐4‐S and collagen type I. For the dataset of the sample and reference spectra, a reference spectrum used in the EMSC was created for each dataset. To analyze the main variation in the FTIR spectra between the two diets, principal component analysis (PCA) was used. The FTIR images were analyzed by in‐house written algorithms in MATLAB (version R2012b, MathWorks Inc, Sherborn, MA). Before quantifying collagen type I and C‐4‐S in FTIR images of skeletal muscle, the FTIR images were segmented into muscle fibers and connective tissue. First, patches within areas of muscle and connective tissue were selected manually. Then the cosine similarity between each pixel spectrum in the image and the mean spectrum of each patch was calculated. Finally, each pixel was designated as muscle fiber or connective tissue by selecting the tissue type having the highest cosine similarity. Muscle spectra were discarded to reduce quantification bias due to unequal sizes of connective tissue bands.

To visualize the estimated concentrations of collagen type I and C‐4‐S, pixel‐wise correlations images were calculated between pre‐processed connective tissue spectra and pre‐processed pure components spectra of collagen type I and C‐4‐S, respectively. The concentrations between low‐ and high‐starch samples are compared by superimposing their respective histograms.

Full MATLAB code and a MATLAB graphical user interface for EMSC with several extensions are freely available at http://nofimaspectroscopy.org.

## 3 RESULTS AND DISCUSSION

The following results present and discuss the potential of spectroscopic analysis of connective tissue in skeletal muscle of Atlantic cod. The results are compared with biochemical results from a previous study 36 and immunostaining.

### 3.1 Pure components results

**Figure 1:**
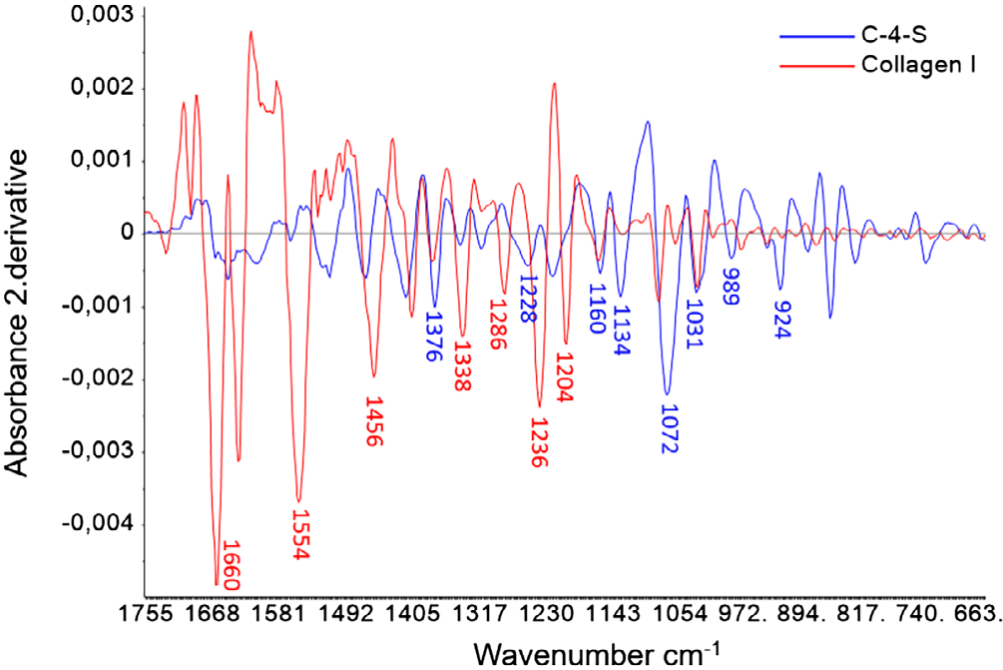
Savitzky‐Golay's second derivative spectra for C‐4‐S (blue) and type I collagen (red) in pure commercial compound in the region 1800 to 800 cm^−1^. The peak assignments are given in Table 1

The pure components, collagen I and C‐4‐S, are chosen because other studies have shown that collagen I is the main collagen in the connective tissue in fish 5 and C‐4‐S is the most prominent GAG present in the connective tissue in cod 38. The FTIR second derivative spectrum of commercial pure component collagen type I shows the strongest bands at amide I (1585‐1720 cm^−1^) and amide II (1500‐1586 cm^−1^) (Figure 1). These bands are typical for proteins and are mainly related to the stretching vibration of peptide C=O bonds, and the combination of C‐N stretching with contribution N‐H bending vibration, respectively 24, 34, 39. The pure component spectrum of C‐4‐S, on the other hand, reveal its strongest band in the carbohydrate region 1140‐985 cm^−1^ mainly caused by from the sugar groups of the side chains and its sulfate group SO_3_^−^ 23, 24, 27, 34, 40. In the second derivative spectra, peak positions are visible as minima. The second derivative bands obtained by FTIR and the literature assignment of the pure components are summarized in Table 1.

**Table 1:** Assignment of second derivative FTIR spectral peaks from pure commercial components collagen type I and C‐4‐S

Wavenumber cm^−1^	Compounds	Assignments	Reference
1660	C=O	Amide I in collagen type I	24, 31, 34, 39
1554	C‐N + N‐H	Amide II in collagen type I	24, 31
1456	CH_2_	Asymmetric bending vibration in collagen type I	23, 24
1376	CH_3_	Symmetric bending vibration of GAGs	40
1338	CH_2_	Side chain vibration of collagen type I	24, 33
1286	CH_2_	Collagen amide III vibrations with CH2 wagging vibration from the glycine backbone and proline sidechain	31
1236	C‐N N‐H CH_2_	Collagen amide III vibration from C‐N stretching, N‐H bending vibrations and wagging vibrations of CH_2_ groups in the glycine backbone and proline side chains.	24
1228	O‐SO_3_^−^	Asymmetric stretching vibration in GAGs	40
1204	CH_2_	Collagen amide III vibrations with CH_2_ wagging vibration from the glycine backbone and proline sidechain	31
1161	C‐O	Stretching vibration of the carbohydrate residues	41
1134	C‐O‐S	Asymmetric stretching of GAGs	40
1082	C‐O	Stretching vibration of the carbohydrate residues in collagen and PGs	31
1072	C‐O‐C, C‐OH and C‐C	Sugar ring of GAGs	24, 40
1060	C‐O/ SO_3_^−^	C‐O stretching vibration of the carbohydrate residues in collagen and PGs/ SO_3_^−^ symmetric stretching vibration of sulfated GAGs	27, 31, 34, 40, 42
1031	C‐O	Stretching vibration of the carbohydrate residues in collagen and PGs	31, 34
989		Not assigned	
924		Sulfate carrying proteoglycan aggrecan	24

Abbreviations: FTIR, Fourier‐transform infrared spectroscopy; GAG, glycosaminoglycan.

**Figure 2:**
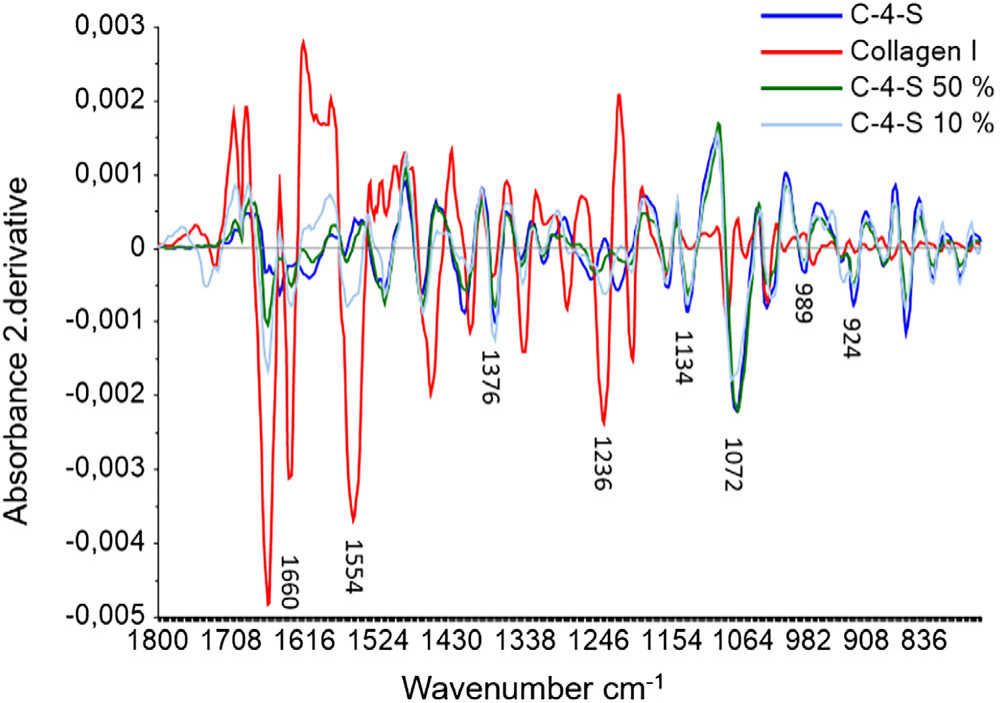
Savitzky‐Golay's second derivative spectra of the pure components C‐4‐S (blue line) and collagen type I (red line), and a mixture of 50% C‐4‐S and 50% collagen type I (green line) and a mixture of 10% C‐4‐S and 90% collagen type I (light blue line). The green and light blue line shows most similarity to collagen type I in the region 1800 to 1200 cm^−1^and to C‐4‐S at the absorbance 1134, 989 and 924 cm^−1^

To mimic the connective tissue content and to explore the spectral properties of collagen type I and C‐4‐S in a combination, the pure components were mixed in different concentrations with each other. In Figure 2, the green line refers to a mixture of 50% collagen type I and 50% C‐4‐S. This mixture shows the highest spectral similarity with collagen type I (red line) at amide I and II and to the C‐4‐S spectrum (blue line) at the bands 1134, 989 and 924 cm^−1^. The light blue line refers to 90% collagen type I and 10% C‐4‐S. This spectral signature is most similar to collagen type I in the region 1800 to 1200 cm^−1^. At 1134, 989 and 924 cm^−1^ this mixture shows a less prominent similarity to the C‐4‐S spectrum, but obvious enough to assume that these peaks are reliable indicators for GAGs. This peak assignment to GAGs is in accordance with previous studies of cartilage tissue done by Camacho et al 23, 24, 43 The peak at 924 cm^−1^ has been described to correspond to aggrecan, a PG mainly consisting of sulfated GAGs 24, 42.

### 3.2 FTIR spectroscopy

**Figure 3:**
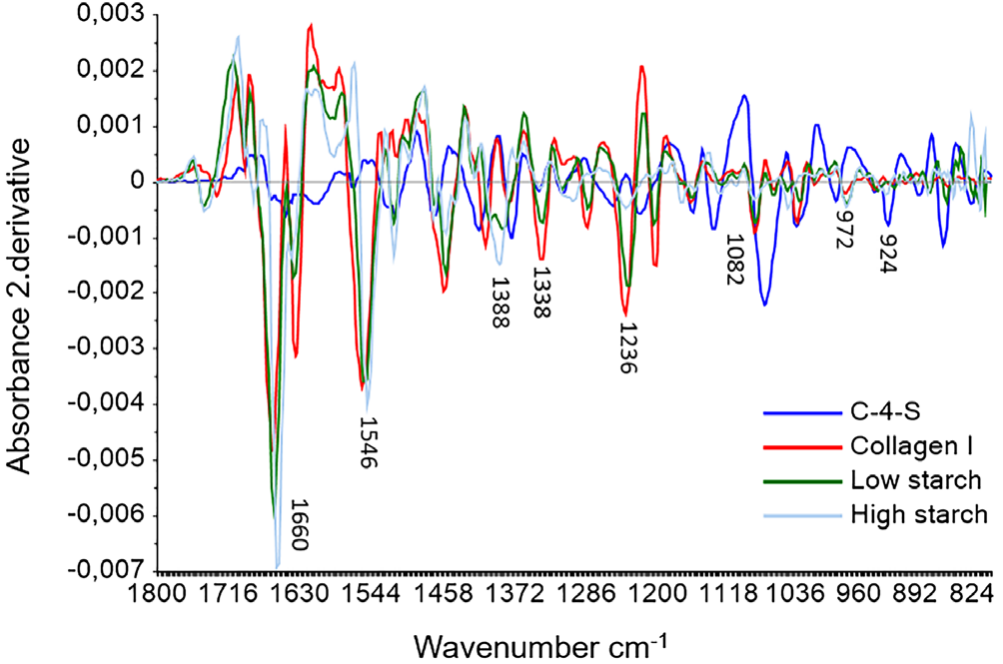
Savitzky‐Golay's second derivative spectra from connective tissue of Atlantic cod fed with low and high starch diet (green and light blue line) compared with the pure components C‐4‐S (blue line) and collagen type I (red line) in the region 1800 to 800 cm^−1^

In Figure 3, Savitzky‐Golay's second derivative FTIR spectra of the connective tissue of cod fed with low and HS diet (green and light blue line) and spectra of the pure components C‐4‐S (blue line) and collagen type I (red line) are shown. The connective tissue spectra are based on the average of three replicates from three different fish from each diet. The major bands obtained from connective tissue are the amide I (1656 cm^−1^) and II (1546 cm^−1^). They are assigned to the stretching vibration of C=O functional groups, a combination of C‐N stretching and N‐H bending vibration in the triple helix of collagens, respectively. The band at 1338 cm^−1^ arises from the CH_2_ side chain vibrations in collagen 24, and the band at 1082 cm^−1^ is related to the C‐O stretching vibrations of the carbohydrate residue present in both collagen and PG molecules 31. The connective tissue spectra are more similar to the pure component collagen type I than the C‐4‐S spectrum (Figure 3). This is expected since collagen is the main component in the connective tissue 44, 45. It is unknown how much GAGs there is in the connective tissue sample and if it is detectable. In the previous section, the pure components were mixed to see how and if the amount of C‐4‐S influenced the collagen type I spectrum. At a concentration of 90% collagen type I and 10% C‐4‐S it was possible to detect the GAGs in the area 1140–985 cm^−1^. In the connective tissue spectra the GAGs peaks at 1134, 989 and 924 cm^−1^ are not prominent, this means that it is too small an amount GAGs of the connective tissue to be visible in the connective tissue spectra.

**Figure 4:**
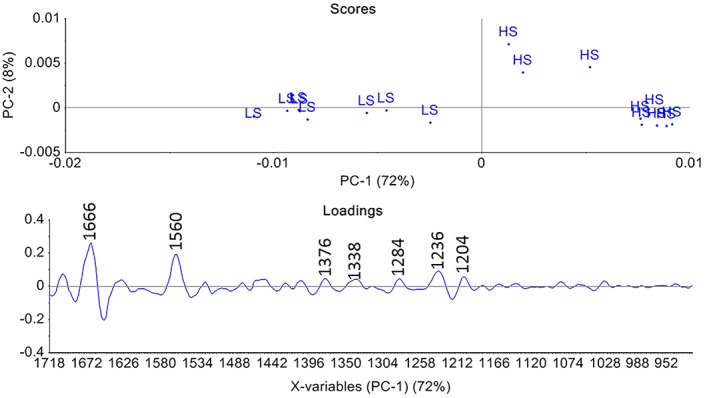
Principal component analysis score and loading plot of connective tissue in Atlantic cod fed with low starch (LS) and high starch (HS) diet in the region of 1718–920 cm^−1^

**Figure 5:**
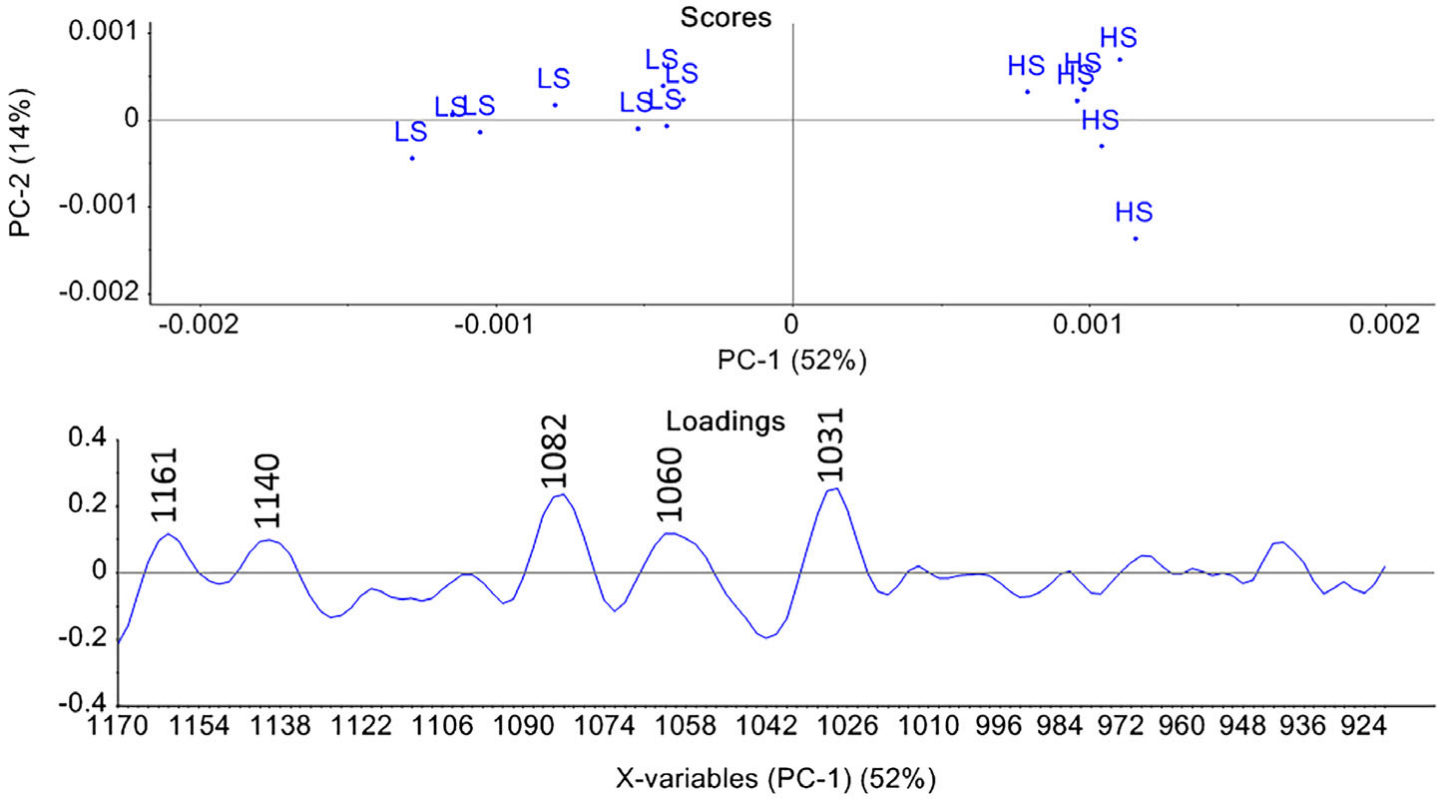
PCA score and loading plot of connective tissue in Atlantic cod fed with low starch (LS) and high starch (HS) diet in the region of 1170 to 920 cm^−1^

PCA score and loading plots obtained from connective tissue spectra of LS and HS fed fish are presented in Figures 4 and 5. In the score plot of the spectral region 1800 to 920 cm^−1^, the fish fed with the LS diet appear to the left and the fish fed with the HS diet to the right. Despite some intra‐group variation, it is possible to distinguish between the two diets by PCA. The main variation between the two groups is along the first Principal Component (PC1), which describes 72% of the variation. The PCA loading describes many of the same features as the pure component collagen type I spectrum. The loading values around 1666, 1546, 1338 and 1236 cm^−1^ are all assigned to collagen. The HS group to the right in the score plot correlates with loadings and seem to have a higher absorbance of collagen type I component. This is in agreement with a previous study 36 of the same material that showed a higher amount of collagen type I by biochemical methods in the fish fed with HS. In the study done by Tingbø (2012) 36, the collagen type I component was measured on mRNA level using real‐time PCR and the collagen type I showed an increased level in the high‐starch group. The differences in collagen content in the connective tissue can be seen by both biochemical methods and spectroscopically.

The PCA of spectral region 1170 to 920 cm^−1^ (Figure 5) was carried out for analyzing GAGs specifically. The loadings of PC 1 explain 52% of the variance between the diets. The bands at 1160, 1134, 1082, 1060 and 1031 cm^−1^ contribute to the separation of the HS and LS groups. The bands at 1161 cm^−1^, 1134 cm^−1^ are found in the pure component spectrum of C‐4‐S. The band 1134 cm^−1^ are assigned to the C–O–S asymmetric stretching vibration in GAGs 40, 46, while the peak at 1161 cm^−1^ is assigned to C‐O stretching vibration of the carbohydrate residues 41. The bands at 1082, 1060 and 1031 cm^−1^ are assigned to the C‐O stretching vibration of the carbohydrate residues in both collagen and PGs 27, 34. 1082, 1060 and 1031 cm^−1^ are quite pronounced in the collagen type I spectrum, and only 1031 cm^−1^ is strong in the C‐4‐S spectrum. The 1060 cm^−1^ has also been assigned to SO_3_‐stretching vibration 27, 40. This band is absent in the pure C‐4‐S spectrum, but existing in the pure collagen type I spectrum. Since 1060 cm^−1^ is also assigned to C‐O stretching vibration of carbohydrate residues in both collagen and PGs 31, it is more trustworthy to assign the band at 1060 cm^−1^ to collagen. The groups are well separated, but since the most prominent peaks are assigned to collagen it is difficult to state the difference according to GAGs. So also in this PCA, the HS group to the right in the score plot correlate to the most prominent loadings which are mainly assigned to collagen.

### 3.3 FTIR imaging spectroscopy and immunohistochemistry

**Figure 6:**
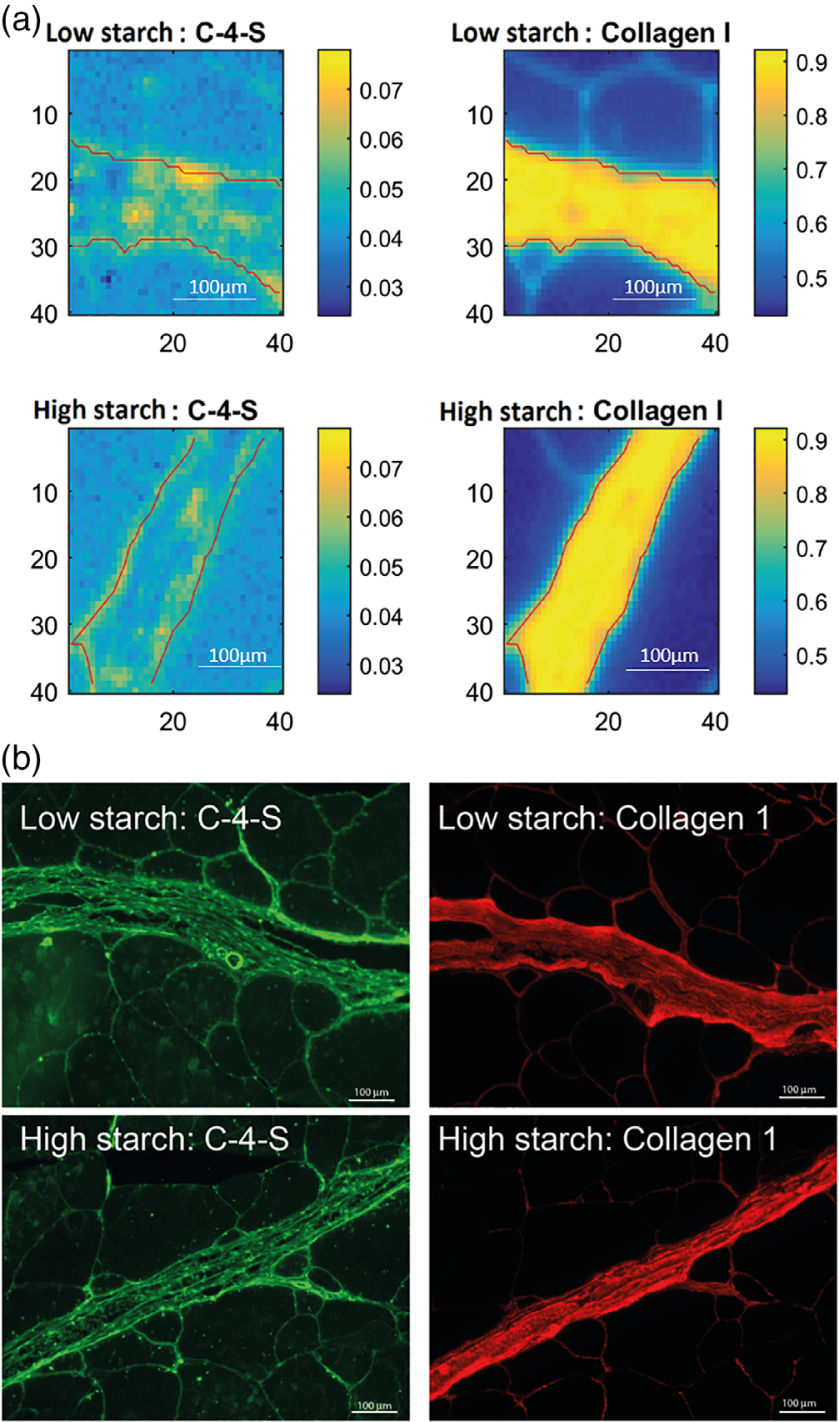
Fourier‐transform infrared spectroscopy (FTIR) and immunohistochemistry images of connective tissue in Atlantic cod fed with low starch (LS) and high starch (HS) diet. The FTIR images (A) are pixel‐wise correlated to the pure component spectra of C‐4‐S and collagen type I, respectively. The FTIR images are compared with immunohistochemistry images stained with C‐4‐S and collagen type I (B)

FTIR imaging was utilized to image the individual components collagen type I and C‐4‐S in the connective tissue in the low and HS fed fish. The segmented connective tissue FTIR images are pixel‐wise correlated to the pure component spectra of collagen type I and C‐4‐S. The FTIR spectral map of collagen type I and C‐4‐S obtained from a thin connective tissue section showed variation in the intensities of the absorbance that arose from C‐4‐S and collagen type I (Figure 6A). It is clear for these images that collagen has the greatest density and is the main component in connective tissue, compared to a much weaker absorbance for C‐4‐S. Immunohistochemistry images were stained with antibodies against collagen type I and C‐4‐S, respectively, and are shown for comparison (Figure 6B) to the FTIR images. For collagen, the FTIR images reveal a collagen distribution that closely resembles the collagen distribution as imaged by immunohistochemistry. It is also well known that collagen is the dominating connective tissue component. 5, 6. Still, the FTIR images (Figure 6A) detect the most C‐4‐S rich regions, as shown by the corresponding immunohistochemistry images (Figure 6B).

**Figure 7:**
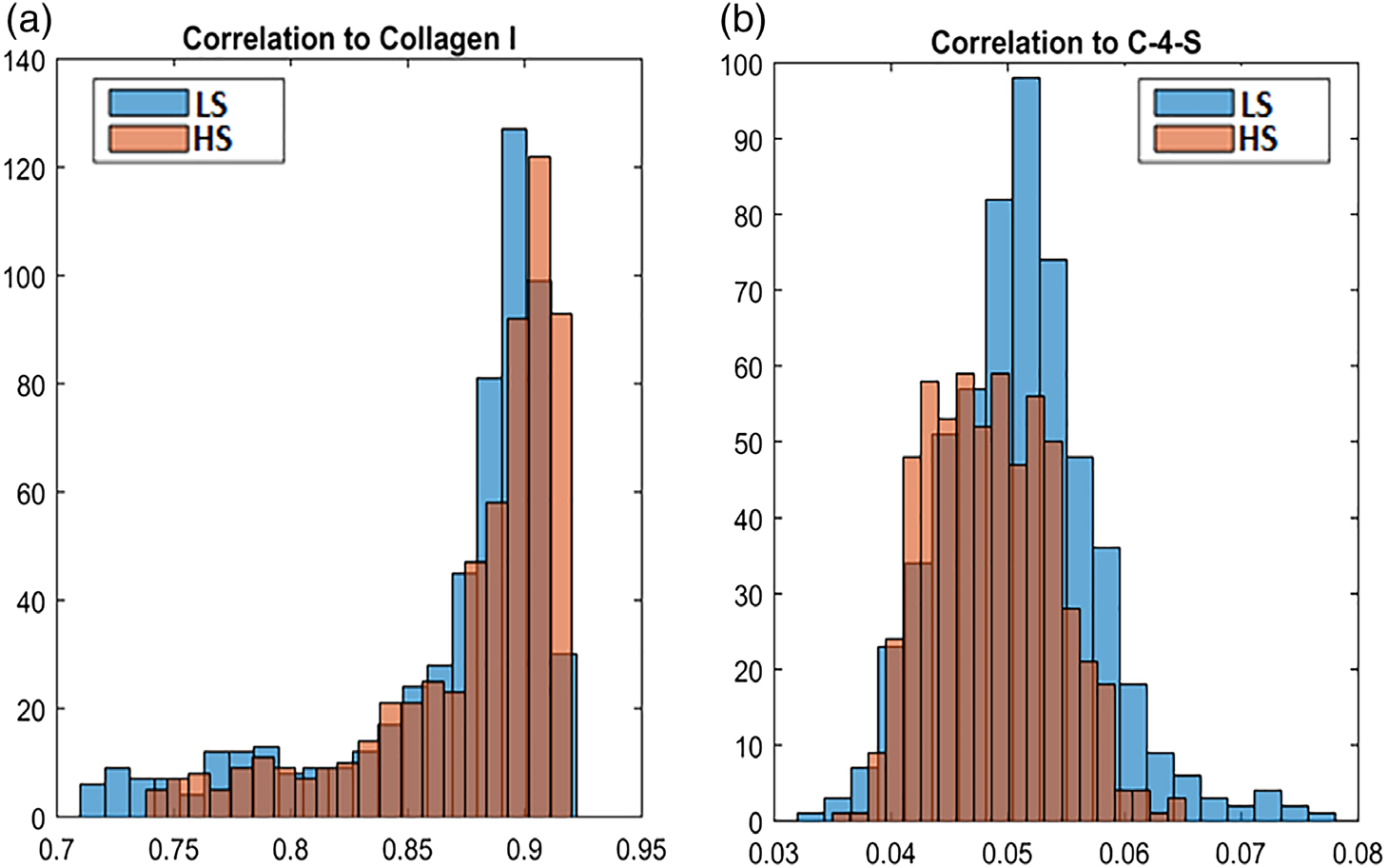
The two diets are compared in the same histogram for correlation to the pure component collagen type I (A) and C‐4‐S (B). For collagen type I, the high starch (HS) diet shows a slightly higher correlation with the pure component collagen type I spectrum than the low starch (LS) diet. For C‐4‐S the LS diet shows a bit higher correlation to the pure component C‐4‐S spectrum than the HS diet

The accompanying histograms of the correlation images are provided in Figure 7. Here, the two diets are compared in the same histogram for each of the two components. As shown in Figure 7A, FTIR‐bands are highly correlated with the pure component collagen type I spectrum. For the correlation to collagen type I, the HS diet shows a slightly higher correlation with the pure component collagen type I spectrum than the LS diet. For the correlation to C‐4‐S, the connective tissue image shows a much weaker correlation to the pure component C‐4‐S. Although; the LS diet seems to correlate slightly better with the pure component C‐4‐S than the HS diet. Both these indications are in contrast to the previous study 36 of the same material.

## 4 CONCLUSION

To conclude, this study shows that important extracellular matrix components for fish filet quality are detectible by FTIR spectroscopy. In the industry, the development of fast and more environmentally friendly techniques that provide a large amount of information is of big interest. By PCA, it was possible to distinguish between the two diets very well according to collagen type I. The pixel‐wise correlation images showed a high correlation to the pure collagen type I spectrum and a weaker correlation to the pure C‐4‐S spectrum. Still, the FTIR images detect the most GAGs rich regions in the connective tissue. The accompanying histograms managed to see the difference in the relative content of both collagen type I and C‐4‐S between the two diets. The study also shows that to investigate small compounds as C‐4‐S together with large molecules as collagen is difficult. For analyzing the relative amount of C‐4‐S, it is needed to correlate the connective tissue spectra with the pure components spectra. Although, these results support the potential of FTIR microspectroscopy and imaging for analyzing biological components in tissue.

## CONFLICT OF INTEREST

The authors declare no potential conflict of interest.

## AUTHOR BIOGRAPHIES

Please see Supporting Information online.

## Supporting information


**Author Biographies**

## References

[1] J. Lavety , O. A. Afolabi , R. M. Love , Int. J. Food Sci. Technol. 1988, 23, 23.

[2] K. H. Eggen , A. Malmstrom , S. O. Kolset , BBA‐Protein Struct. M. 1994, 1204, 287.

[3] K. E. Kadler , A. Torreblanco , E. Adachi , B. E. Vogel , Y. Hojima , D. J. Prockop , Biochemistry 1991, 30, 5081. 2036375 10.1021/bi00234a035

[4] J. Myllyharju , K. I. Kivirikko , Ann. Med. 2001, 33, 7. 11310942 10.3109/07853890109002055

[5] D. A. Bruggemann , M. A. Lawson , J. Fish Biol. 2005, 66, 810.

[6] K. Sato , R. Yoshinaka , Y. Itoh , M. Sato , Comp. Biochem. Physiol. B 1989, 92, 87. 10.1016/0305-0491(88)90053-3 3396321

[7] L. Kjellen , U. Lindahl , Annu. Rev. Biochem. 1991, 60, 443. 1883201 10.1146/annurev.bi.60.070191.002303

[8] A. M. Cribb , J. E. Scott , J. Anat. 1995, 187, 423. 7592005 PMC1167437

[9] S. G. Velleman , X. S. Liu , K. H. Eggen , K. E. Nestor , Poult. Sci. 1999, 78, 1619. 10560838 10.1093/ps/78.11.1619

[10] Ø. Hagen , C. Solberg , E. Sirnes , I. A. Johnston , J. Agric. Food Chem. 2007, 55, 5803. 17567023 10.1021/jf063614h

[11] Ø. Hagen , C. A. Johnsen , Food Chem. 2016, 190, 786. 26213039 10.1016/j.foodchem.2015.06.007

[12] J. D. Esko , K. Kimata , U. Lindahl , Essentials of Glycobiology, 2nd ed., Cold Spring Harbor Laboratory Press, Cold Spring Harbor, NY 2009. 20301239

[13] W. D. Comper , T. C. Laurent , Physiol. Rev. 1978, 58, 255. 414242 10.1152/physrev.1978.58.1.255

[14] M. G. Tingbo , S. O. Kolset , R. Ofstad , G. Enersen , K. O. Hannesson , Comp. Biochem. Physiol. B Biochem. Mol. Biol. 2006, 143, 441. 16459119 10.1016/j.cbpb.2005.12.022

[15] M. G. Tingo , S. O. Kolset , R. Ofstad , G. Enersen , K. O. Hannesson , Comp. Biochem. Physiol. B Biochem. Mol. Biol. 2005, 140, 349. 15694582 10.1016/j.cbpc.2004.09.032

[16] J. S. Torgersen , E. O. Koppang , L. H. Stien , A. Kohler , M. E. Pedersen , T. Morkore , PloS One 2014, 9, e85551. 24416425 10.1371/journal.pone.0085551 PMC3887068

[17] A. Jonsson , S. Sigurgisladottir , H. Hafsteinsson , K. Kristbergsson , Aquac. Nutr. 2001, 7, 81.

[18] M. A. Pratta , W. Q. Yao , C. Decicco , M. D. Tortorella , R. Q. Liu , R. A. Copeland , R. Magolda , R. C. Newton , J. M. Trzaskos , E. C. Arner , J. Biol. Chem. 2003, 278, 45539. 12890681 10.1074/jbc.M303737200

[19] M. J. Baker , J. Trevisan , P. Bassan , R. Bhargava , H. J. Butler , K. M. Dorling , P. R. Fielden , S. W. Fogarty , N. J. Fullwood , K. A. Heys , C. Hughes , P. Lasch , P. L. Martin‐Hirsch , B. Obinaju , G. D. Sockalingum , J. Sule‐Suso , R. J. Strong , M. J. Walsh , B. R. Wood , P. Gardner , F. L. Martin , Nat. Protoc. 2014, 9, 1771. 24992094 10.1038/nprot.2014.110 PMC4480339

[20] G. Socrates,* Infrared Characteristic Group Frequencies, *John Wiley & Sons, New York 1994.

[21] X. Bi , X. Yang , M. P. G. Bostrom , D. Bartusik , S. Ramaswamy , K. W. Fishbein , R. G. Spencer , N. P. Camacho , Anal. Bioanal. Chem. 2007, 387, 1601. 17143596 10.1007/s00216-006-0910-7 PMC2944229

[22] L. Rieppo , S. Saarakkala , T. Narhi , J. Holopainen , M. Lammi , H. J. Helminen , J. S. Jurvelin , J. Rieppo , Microsc. Res. Tech. 2010, 73, 503. 19839035 10.1002/jemt.20789

[23] A. Boskey , N. P. Camacho , Biomaterials 2007, 28, 2465. 17175021 10.1016/j.biomaterials.2006.11.043 PMC1892909

[24] N. P. Camacho , P. West , P. A. Torzilli , R. Mendelsohn , Biopolymers 2001, 62, 1. 11135186 10.1002/1097-0282(2001)62:1<1::AID-BIP10>3.0.CO;2-O

[25] B. d. C. Vidal , M. L. S. Mello , PloS One 2016, 11, e0151989. 10.1371/journal.pone.0151989 PMC4807954 27015280

[26] L. Rieppo , S. Saarakkala , J. S. Jurvelin , J. Rieppo , J. Biomech. 2013, 46, 1269. 23538002 10.1016/j.jbiomech.2013.02.022

[27] L. Rieppo , T. Narhi , H. J. Helminen , J. S. Jurvelin , S. Saarakkala , J. Rieppo , J. Biomed. Opt. 2013, 18, 097006. 24064950 10.1117/1.JBO.18.9.097006

[28] L. Rieppo , J. Rieppo , J. S. Jurvelin , S. Saarakkala , Plos One 2012, 7, e32344. 22359683 10.1371/journal.pone.0032344 PMC3281137

[29] S. Saarakkala , P. Julkunen , Cartilage 2010, 1, 262. 26069557 10.1177/1947603510368689 PMC4297054

[30] M. Kim , X. H. Bi , W. E. Horton , R. G. Spencer , N. P. Camacho , J. Biomed. Opt. 2005, 10, 031105. 16229630 10.1117/1.1922329

[31] M. Jackson , L. P. Choo , P. H. Watson , W. C. Halliday , H. H. Mantsch , BBA‐Mol. Basis Dis. 1995, 1270, 1. 10.1016/0925-4439(94)00056-v 7827129

[32] A. Savitzky , M. J. E. Golay , Anal. Chem. 1964, 36, 1627.

[33] L. Rieppo , S. Saarakkala , T. Narhi , H. J. Helminen , J. S. Jurvelin , J. Rieppo , Osteoarthr. Cartil. 2012, 20, 451. 22321720 10.1016/j.joca.2012.01.010

[34] A. Kohler , D. Bertrand , H. Martens , K. Hannesson , C. Kirschner , R. Ofstad , Anal. Bioanal. Chem. 2007, 389, 1143. 17639358 10.1007/s00216-007-1414-9

[35] B. Zimmermann , A. Kohler , Appl. Spectrosc. 2013, 67, 892. 23876728 10.1366/12-06723

[36] M. G. Tingbo , M. E. Pedersen , F. Grondahl , S. O. Kolset , E. Veiseth‐Kent , G. Enersen , K. O. Hannesson , Fish Shellfish Immunol. 2012, 33, 582. 22789715 10.1016/j.fsi.2012.06.025

[37] S. B. Rønning , T.‐K. Østbye , A. Krasnov , T. T. Vuong , E. Veiseth‐Kent , S. O. Kolset , M. E. Pedersen , Fish Physiol. Biochem. 2017, 43, 549. 27807712 10.1007/s10695-016-0309-0 PMC5374190

[38] K. O. Hannesson , M. G. Tingbo , R. L. Olsen , G. Enersen , A. B. Baevre , R. Ofstad , Comp. Biochem. Physiol. B Biochem. Mol. Biol. 2007, 146, 512. 17270478 10.1016/j.cbpb.2006.11.024

[39] B. De Campos Vidal , M. L. S. Mello , Micron 2011, 42, 283. 21134761 10.1016/j.micron.2010.09.010

[40] R. Servaty , J. Schiller , H. Binder , K. Arnold , Int. J. Biol. Macromol. 2001, 28, 121. 11164228 10.1016/s0141-8130(00)00161-6

[41] H. P. Wang , H. C. Wang , Y. J. Huang , Sci. Total Environ. 1997, 204, 283. 9335161 10.1016/s0048-9697(97)00180-0

[42] A. Hanifi , H. McCarthy , S. Roberts , N. Pleshko , PloS One 2013, 8, e64822. 23717662 10.1371/journal.pone.0064822 PMC3661544

[43] N. P. Camacho , P. Carroll , C. L. Raggio , Calcif. Tissue Int. 2003, 72, 604. 12574874 10.1007/s00223-002-1038-1

[44] K. Gelse , E. Poschl , T. Aigner , Adv. Drug Deliv. Rev. 2003, 55, 1531. 14623400 10.1016/j.addr.2003.08.002

[45] S. Ricard‐Blum , F. Ruggiero , Pathologie Biologie. 2005, 53, 430. 16085121 10.1016/j.patbio.2004.12.024

[46] S. M. Bychkov , S. A. Kuzmina , Bull. Exp. Biol. Med. 1991, 112, 1571.

