# Supplementary material for: The use of Fourier‐transform infrared spectroscopy to characterize connective tissue components in skeletal muscle of Atlantic cod (Gadus morhua L.)
Source: J Biophotonics. 2019 Jul 1;12(9):e201800436. doi: 10.1002/jbio.201800436 (PMC7065610; doi:10.1002/jbio.201800436)
Supplement: Supplementary file 1 — Author Biographies [file JBIO-12-e201800436-s001.docx]

| 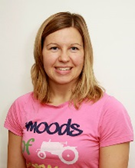 | **Karen Wahlstrøm Sanden** holds a master’s degree in food science from Norwegian University of Life Sciences, Ås, Norway. She is currently working on her Ph.D. at the Norwegian Institute of Food, Fisheries and Aquaculture Research focusing on FT-IR spectroscopy in skeletal muscles. Her main interest is the structure of connective tissue in fish and meat. |
| --- | --- |
| 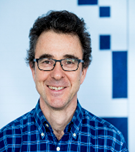 | **Achim Kohler** obtained his Ph.D. in theoretical physics in 1998. After his Ph.D. he has been working for almost 15 years at Nofima, The Norwegian Institute of Food Fisheries and Aquaculture Research in Norway, where he developed vibrational spectroscopy techniques for food quality and safety analysis. He is currently a professor in physics at the Faculty of Science and Technology at the Norwegian University of Life Sciences, in Ås, Norway. Prof. Kohler and his BioSpec group have been keen on understanding and modeling of scattering and absorption in vibrational spectroscopy since almost two decades. |
| 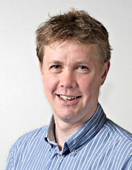 | **Nils Kristian Afseth** holds a master’s degree in organic chemistry from the Norwegian University of Technology and Science, Trondheim. In 2007 he received his Ph.D. in biospectroscopy from the Norwegian University of life sciences, Ås. He is currently working as a senior research scientist at Nofima, the Norwegian Institute of food, fisheries and aquaculture research. His research involves vibrational spectroscopy and chemometrics for rapid analysis of food, food components and biological systems. |
| 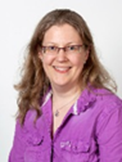 | **Ulrike Böcker** holds a master’s degree in food science and Technology from the University of Hohenheim, Stuttgart, Germany. In 2008 she received a Ph.D. in Food Chemistry awarded by the Norwegian University of Life Sciences. She is currently working at the Norwegian Institute of Food, Fisheries and Aquaculture Research focusing on FT-IR and Raman (micro-) spectroscopy in the field of food science, both as methods for investigation process-induced effects on protein structure and as rapid quality screening methods compared to standard laboratory methods. |
| 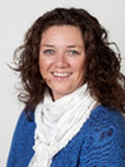 | **Sissel Beate Rønning** holds a civil engineer degree in Gene technology from the University of Tromsø, Tromsø, Norway and has a Ph.D. on GMOs from the National Veterinary Institute. After finished her Ph.D. she worked as a Post Doc studying cancer cell biology and medical research at Institute for Clinical Medicine, Oslo University Hospital. She then took a second Post Doc at Nofima As, establishing a primary muscle cell model system. She is now a research scientist at Nofima, responsible for in vitro cell model systems. Her research involves muscle development, protein production, biomaterials, extracellular matrix, histology, food quality, bioactivity, food and health. |
| 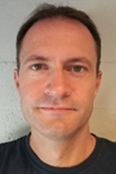 | **Kristian Hovde Liland** holds a Master of Science and Ph.D., both in Applied Statistics from the Norwegian University of Life Sciences, Ås, Norway. He is currently working as an Associate Professor in Data Science at the Norwegian University of Life Sciences. His research includes method development and application of spectral background correction and multivariate data analysis. |
| 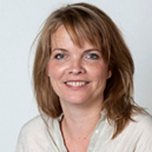 | **Mona E. Pedersen** received her Ph.D. in Biochemistry from University of Oslo (UiO), Oslo, Norway in 2001. The topic of her thesis was the role of connective tissue and meat quality. At present she is working as a Research Scientist at the Norwegian Institute of Food, Fisheries and Aquaculture research. She has since her Ph.D. and Post-docs periods been working with extracellular matrix related to skeletal muscle growth and bone development. Her research involves histology techniques, molecular and biochemical methods for characterization of extracellular matrix production and turnover in tissues. She also uses in vitro cell systems to reveal mechanisms involved. |
